# Cellulases: key properties, natural sources, and industrial applications

**DOI:** 10.18699/vjgb-25-141

**Published:** 2025-12

**Authors:** A.V. Zadorozhny, N.M. Slynko, S.V. Bannikova, N.V. Bogacheva, V.N. Shlyakhtun, A.R. Vasilieva, E.Yu. Bukatich, V.S. Ushakov, Yu.E. Uvarova, A.V. Korzhuk, A.A. Shipova, D.V. Bochkov, E.Y. Pavlova, D.O. Chesnokov, S.E. Peltek

**Affiliations:** Institute of Cytology and Genetics of the Siberian Branch of the Russian Academy of Sciences, Novosibirsk, Russia; Institute of Cytology and Genetics of the Siberian Branch of the Russian Academy of Sciences, Novosibirsk, Russia; Institute of Cytology and Genetics of the Siberian Branch of the Russian Academy of Sciences, Novosibirsk, Russia; Institute of Cytology and Genetics of the Siberian Branch of the Russian Academy of Sciences, Novosibirsk, Russia; Institute of Cytology and Genetics of the Siberian Branch of the Russian Academy of Sciences, Novosibirsk, Russia; Institute of Cytology and Genetics of the Siberian Branch of the Russian Academy of Sciences, Novosibirsk, Russia; Institute of Cytology and Genetics of the Siberian Branch of the Russian Academy of Sciences, Novosibirsk, Russia; Institute of Cytology and Genetics of the Siberian Branch of the Russian Academy of Sciences, Novosibirsk, Russia; Institute of Cytology and Genetics of the Siberian Branch of the Russian Academy of Sciences, Novosibirsk, Russia; Institute of Cytology and Genetics of the Siberian Branch of the Russian Academy of Sciences, Novosibirsk, Russia; Institute of Cytology and Genetics of the Siberian Branch of the Russian Academy of Sciences, Novosibirsk, Russia; Institute of Cytology and Genetics of the Siberian Branch of the Russian Academy of Sciences, Novosibirsk, Russia; Institute of Cytology and Genetics of the Siberian Branch of the Russian Academy of Sciences, Novosibirsk, Russia; Institute of Cytology and Genetics of the Siberian Branch of the Russian Academy of Sciences, Novosibirsk, Russia; Institute of Cytology and Genetics of the Siberian Branch of the Russian Academy of Sciences, Novosibirsk, Russia

**Keywords:** celulase, fungal and bacterial cellulases, extremophilic cellulases, celulase structure and essential properties, cellulase complex, biotechnology, genetic engineering, целлюлаза, грибные и бактериальные целлюлазы, экстремофильные целлюлазы, структура и основные свойства целлюлаз, целлюлазный комплекс, биотехнология, генная инженерия

## Abstract

This review focuses on cellulases, a subclass of hydrolases that catalyse the breakdown of the polysaccharide cellulose. Cellulases are of immense practical significance, given that cellulose-containing materials are utilised across a multitude of industrial sectors. An overview of the fundamental properties and structure of cellulases is provided. However, primary attention is paid to the industrial application of these enzymes, with other aspects discussed within this context. The most practically significant bacterial and fungal cellulases are analysed, with their key benefits and differences being emphasised. Particular attention is paid to extremophilic (specifically thermo-, psychro-, and halophilic) cellulases, as they possess properties essential for modern technological processes. Given that practical application necessitates mass production and an optimal combination of enzymatic characteristics, the creation of effective producers and the modification of cellulase properties are also assessed. Finally, key trends in cellulase production approaches and their future application potential are summarised.

## Abstract (ru)

В последние годы целлюлаза привлекает огромное внимание как промышленно важный фермент с широким спектром применения. Целлюлазы представляют собой сложную группу ферментов, которые секретируются многообразием микроорганизмов, включая грибы и бактерии. Они относятся к подклассу ферментов гидролаз, субстратом которых является полисахарид целлюлоза. Целлюлазы имеют огромное практическое значение, поскольку содержащие целлюлозу материалы используются во множестве отраслей народного хозяйства. В этом обзоре приведены сведения об основных свойствах и структуре целлюлаз. Но основное внимание уделено применению этих ферментов в промышленности, а прочие аспекты, так или иначе, рассматриваются с учетом этого. Исследованы имеющие наибольшее практическое значение бактериальные и грибные целлюлазы, их основные преимущества и отличия. Отдельно рассмотрены экстремофильные (а именно термо-, психро- и галофильные) целлюлазы как обладающие свойствами, нужными в условиях современных технологических процессов. Поскольку для практического применения необходимы массовая продукция и оптимальное сочетание свойств ферментов, внимание также уделено получению эффективных продуцентов и модификации свойств, производимых целлюлаз. Наконец, обозначены ключевые тенденции в подходах к производству целлюлаз и перспективы практического применения.

## Introduction

The rise in environmental consciousness and progress in biotechnology have facilitated the replacement of numerous chemical procedures by enzymatic biocatalysts in a variety of industries, including textiles, leather, pulp and paper, fruit and vegetable processing, food processing, and feed production (Wackett, 2019). Enzymes can be isolated from various sources: animal tissues, plants, and microbial cells. Microbial proteins are frequently more stable than enzymes of similar specificity derived from plant or animal sources and can often be stored under less-than-ideal conditions for extended periods without significant loss of biological activity (Singhania et al., 2010). Most commercial enzymes are obtained from microorganisms. These enzymes are strong candidates for efficient biotechnological processes due to properties like thermostability, broad pH range stability, and multifunctionality, which allow them to function under various physicochemical conditions

Cellulases represent the second largest group of industrial enzymes by market share, and their usage continues to grow alongside increasing demand from a wide range of sectors: food, pulp and paper, textiles, pharmaceuticals, detergents, animal feed, biofuels, and waste management (Ranjan et al., 2023).

Cellulases, similar to other enzymes, are sourced from plants, microorganisms, and animals. Fungi and bacteria are most often used in the production of these enzymes because of traits such as considerable yield and cost reduction. Historically, the principal producers of cellulases have been natural strains of fungi, and their productivity has been enhanced through selection and/or mutagenesis methods. However, the advent and development of recombinant DNA technology, genetic engineering, protein engineering, directed evolution, omics technologies, and high-throughput sequencing have led to the discovery of novel microbes and enzymes for industrial applications (Patel et al., 1994; Kirk et al., 2002; Rubin-Pitel, Zhao, 2006) alongside the development of various recombinant microbial cellulases. These enzymes are of particular significance due to their specific attributes, including cost-effectiveness, energyefficient catalytic processes, environmental compatibility, non-toxicity, and high efficiency.

In numerous scenarios, it is advantageous to use cellulases that exhibit extremophilic properties, including stability and efficiency across a range of temperatures, pH levels, pressures, or in the presence of organic solvents, detergents, and elevated ionic strengths within the operational environment. Enzymes demonstrating these attributes are typically discovered in research on microorganisms within suitable environments, whereas enzymes with uncommon properties are also observed in mesophilic organisms. The increasing demand has led to a gradual expansion in the selection of cellulases with a wide range of properties. The applica‑ tion of enzymes, which are stable under extreme conditions, will optimise outcomes while minimising enzyme consumption.

## Cellulases: key properties and structure

Fungi and bacteria are both employed in the production of various cellulases. Fungi have long been a primary focus due to their ability to secrete significant quantities of enzymes. The recent shift towards bacteria is attributed to their rapid growth rates, multifunctional enzymes, and their ubiquity across diverse ecological niches. Bacteria are not only capable of surviving in harsh conditions but frequently produce stable enzymes that can accelerate catalytic processes more effectively than their fungal counterparts.

Cellulose, a natural polymer, serves as the substrate for cellulase. Cellulases are enzymatic systems that hydrolyse β-1,4-glycoside bonds found within cellulose polymers and its derivatives, yielding soluble oligosaccharides and glucose monomers (Nishida et al., 2007). The biochemical degradation of the cellulose molecule by microorganisms is catalysed by an extracellular enzymatic system comprising three key components (Bhat M.K., Bhat S., 1997). These are: (1) β-1,4-glucan glucanohydrolase (endoglucanase; EC 3.2.1.4), which cleaves the long cellulose chain into shorter fragments; (2) β-1,4-glucan cellobiohydrolase (exoglucanase; EC 3.2.1.91), which acts upon the non-reducing end of the cellulose chain; and (3) β-1,4-glucosidase (EC 3.2.1.21), which breaks the glycosidic bonds of cellobiose and cellodextrins, producing glucose molecules that can readily permeate the cell. In nature, the synergistic action of all three enzymes is required for complete hydrolysis of the cellulose polymer to glucose units (Uhlig, 1998).

The complex spatial structure of the cellulase complex facilitates the positioning of cellulose fibers near the active sites of the enzymes. Cellulase systems are categorised into two distinct types. The first type is exemplified by the extracellular cellulases of filamentous fungi and aerobic bacteria, which act synergistically to decompose cellulose. The second type, found in anaerobic clostridia, attaches to the bacterial cell surface, known as the “cellulosome”.

A typical characteristic of cellulases is a two-domain structure, which includes a catalytic domain and a cellulosebinding domain (CBD), also referred to as a carbohydratebinding module (CBM), which are usually connected by a peptide linker. The active site is found in the catalytic domain, with the CBD ensuring accurate cellulose fiber placement (Mathew et al., 2008).

Cellulose-degrading aerobic fungi, such as Hypocrea jecorina (Trichoderma reesei), produce an enzyme complex that acts synergistically and displays all three primary activities: endoglucanase, exoglucanase, and β-glucosidase (Dashtban et al., 2011; Mukherjee et al., 2012). The process begins with endoglucanases cleaving cellulose fibers in the more amorphous regions. Subsequently, exoglucanases can cleave cellobiose molecules from the more crystalline sections of the fibre that were previously inaccessible. Finally, β-glucosidases hydrolyse the cellobiose

Most anaerobic microorganisms have their cellulosolytic system organised differently. The whole complex of various cellulases and hemicellulases is integrated into a multiprotein complex, the cellulosome, that is attached to the external part of the cell through the non-catalytic protein scaffoldin. The molecular weight of cellulosomes can reach several thousand kDa (Pinheiro et al., 2009). The surface of a single Clostridium thermocellum cell (the most extensively studied cellulolytic bacterium capable of rapid growth on cellulosic substrates) hosts multiple cellulosomes. This ensures the secure attachment of the microorganism to the cellulose fibre, while the spatial arrangement of the various enzymes within the cellulosome facilitates their access to the substrate. Furthermore, clostridia also produce free cellulases not associated with cellulosomes. The presence of a synergistic effect with cellulosomes remains undetermined (Doi, Tamaru, 2001).

This organisational structure of the cellulosolytic system results in simple sugars being produced in the immediate vicinity of the microorganism, significantly facilitating nutrient uptake. In certain scenarios, anaerobic bacteria were observed to regulate cellulosome binding on the cell surface, resulting in free cellulosomes being detected, and to alter the production of various cellulosome components depending on growth conditions (Mohand-Oussaid et al., 1999).

Besides including the complex of cellulolytic enzymes, the cellulosome also comprises carbohydrate fibrils, which can account for up to 90 % of the molecular mass of the cellulosome. They promote the sorption of the enzyme onto the substrate and the sliding of the enzyme along the fibrillar structures of cellulose. Furthermore, the carbohydrate portion protects the protein from the action of denaturing agents and proteases.

The efficacy of different cellulase complexes in cellulose hydrolysis varies significantly when the substrate possesses high crystallinity. A significant reduction in activity occurs in numerous complexes when crystallinity reaches 60–70 %, with hydrolysis limited to the amorphous portion of the substrate. The effectiveness of these complexes in hydrolysing cellulose with high crystallinity is contingent upon the presence of endoglucanases capable of strong adsorption. Consequently, the rate of crystalline cellulose hydrolysis is directly influenced by the quantity of endoglucanase adsorbed.

## Industrial applications of cellulases

Cellulases are in high demand across a wide range of industrial sectors. In the food industry, cellulase is traditionally employed for the extraction and clarification of juices (Azman et al., 2021; Ozyilmaz, Gunay, 2023). In addition, it allows the nutritional characteristics of brown rice to be enhanced. Q. Zhang et al. (2019) demonstrated that cyclic treatment with cellulase combined with germination is an effective method for increasing GABA levels in brown rice while improving its culinary and sensory qualities.

Additionally, cellulase is employed to improve dough quality (Hu X. et al., 2022). Adding potato to wheat flour increases the nutritional content of bread. However, the adverse effects caused by the high dietary fibre content of potato flour can impair gluten matrix formation. The addition of cellulase and/or pectinase promotes loaf volume and softness.

The application of cellulase for oil extraction in the food and cosmetic industries also holds promise. D. Yu et al. (2022) introduced a method for extracting oil from rice bran using magnetically immobilised cellulase in combination with magnetically immobilised alkaline protease. Similarly, M.O. Chiwetalu et al. (2022) presented an extraction method for obtaining fat from Pycnanthus angolensis seeds via pre-treatment with an enzyme from Aspergillus niger strain BC23.

Significant progress has recently been made in lignocellulose biorefinery technologies for the production of biochemicals and biofuels, using cellulase enzymes as a key component (Sharma et al., 2023).

A. Shankar et al. (2024) pre-treated rice straw, rice bran, wheat straw, wood chips, sorghum bagasse, and cotton stalks with the basidiomycete fungus Ganoderma lucidum. Due to its laccase activity, the fungus significantly degraded the lignin present in the biomass. The saccharification of pretreated biomass using cellulase consortia, specifically those isolated from Aspergillus flavus MDU-5 and Trichoderma citrinoviride MDU-1, resulted in an approximate 70 % increase in saccharide yield and an 89 % increase in ethanol yield. Additionally, H. Sha et al. (2023) developed a novel approach to the anaerobic digestion of corn straw using magnetic cellulase coated with nickel and graphite. This system increased methane production by approximately 74 % compared to an uncoated system. Additionally, an increase in the combined population of electroactive Bacteroidota and Methanomicrobiales and improved energy conversion efficiency by up to 57 % were reported.

E. Zanuso et al. (2022) demonstrated the efficacy of using cellulase immobilised on magnetic nanoparticles for the hydrolysis of corn cob biomass. Moreover, the magnetic properties of the carrier make this method promising for continuous operation, contributing to reduced overall process costs.

In the pulp and paper industry, cellulase is used in wastepaper recycling (deinking) and the pre-treatment of raw wood materials for pitch removal (Chutani, Sharma, 2016; Lehr et al., 2021; Singh A. et al., 2021), thereby reducing the environmental impact of the industry.

In the pharmaceutical industry, cellulase is employed to extract biologically active compounds from plant raw materials (Puri et al., 2012; Cao et al., 2019; Hu Y. et al., 2021). It is also widely used in the production of enzyme tablet formulations intended to facilitate the digestion of plant-based foods rich in fiber. VeganZyme, for example, is used both to enhance digestion and to manage metabo- lic disorders (Ranjan et al., 2023). Furthermore, cellulase and papain are used to treat phytobezoars (Iwamuro et al., 2014).

Chemical decomposition and incineration are the most frequently employed techniques for the remediation of environmental pollutants. However, the application of microbial enzymes for purification offers a more straightforward and eco-friendly method (Okino-Delgado et al., 2019). Given that Bacillus cereus and B. subtilis can produce cellulase and lipase, enzymes known for their efficacy in waste degradation, these bacteria were identified by as potentially useful in treating palm oil mill waste (Ranjan et al., 2023).

The study by J. Luo et al. (2021) used cellulase in the simultaneous fermentation of sewage sludge and paper waste, resulting in the production of volatile fatty acids. All these findings collectively suggest the applicability of cellulase for wastewater treatment and its potential for producing value-added products.

Cellulase finds broad application in the manufacture of animal feed. Cellulase and hemicellulase facilitate the hydrolysis of low-protein feeds and β-glucans, consequently improving the nutritional characteristics of the feed. The application of cellulase also allows duodenal viscosity to be reduced and feed consistency to be improved, consequently enhancing digestion and nutrient uptake by animals (Azzaz et al., 2021; Selzer et al., 2021). In poultry farming, the use of cellulase is a viable strategy as it degrades cellulosic bonds, releasing nutrients such as glucose, increasing the energy value of the diet, and thus improving bird performance (Perim et al., 2024).

In the textile industry, cellulase is used to improve the fabric product finish by modifying protruding fibres. Cellulase treatment reduces fabric roughness, increasing smoothness, gloss, and colour brightness (Karmakar, Ray, 2011; Sajith et al., 2016).

The inclusion of cellulase in detergents, alongside proteases and lipases, is intended to enhance washing efficacy. It helps maintain the shape and colour of laundered garments by reducing the formation of fuzz and pills. Additionally, the enzyme contributes to the soil and stain removal process by selectively acting on cellulose fibres from the interior. The cellulase enzyme breaks the bonds between cellulose and dirt particles and alters the fibre surface structure, thereby facilitating soil removal by other detergent ingredients. Currently, both broad-temperature-range (30–60 °C) and mesophilic cellulases are added to detergents (Kasana, Gulati, 2011). Cellulase enzymes are used in detergent compositions to provide cleaning, softening, and colour retention. However, the use of most cellulases has been limited due to the potential negative impact on fabric tensile strength resulting from the hydrolysis of crystalline cellulose. Recently, cellulases with high specificity towards amorphous cellulose have been developed to exploit their cleaning potential without undesirable loss of fabric strength.

## Fungal cellulases

Cellulolytic fungi of the genus Trichoderma have long been considered the premier source of cellulases (Reese, Mandels, 1963). However, the primary bottleneck associated with Trichoderma cellulases is the very low β-glucosidase activity in culture supernatants, coupled with product inhibition of this enzyme (Pérez et al., 2002). Conversely, cellulases produced by the thermophilic fungi Sporotrichum thermophile and Talaromyces emersonii exhibit activity comparable to that of the mesophilic fungus H. jecorina (Coutts, Smith, 1976; Folan, Coughlan, 1978).

Humicola insolens demonstrates exceptional production capabilities of pH-neutral, thermostable cellulases that are industrially relevant (Xu X. et al., 2016).

P. Chellapandi and H.M. Jani investigated the endoglucanase activity of 26 Streptomyces strains isolated from garden soil (Chellapandi, Jani, 2008). The two most promising isolates, selected for their potential celluloly- tic activity on Bennett’s agar medium, were assessed under varying conditions, including carbon and nitrogen sources, and growth conditions. Maximum endoglucanase activity (11.25–11.90 U/ml) was achieved after 72–88 hours in a fermentation medium containing Tween-80, followed by the utilisation of phosphate sources. Both cellulolytic Streptomyces isolates produced nearly identical quantities of enzyme in all trials. However, the influence of media ingredients on endoglucanase induction differed somewhat between the strains. Like mesophilic fungi, thermophilic fungi produce all components of the cellulase complex, synergistically degrading cellulose and hemicellulose (Mathew et al., 2008).

Fungi are a source of vital extracellular enzymes used in industrial applications. Genera such as Trichoderma, Penicillium, and Aspergillus are especially recognised in this regard (Zhao C.H. et al., 2018). In addition, fungi are widely used in the production of industrial cellulases because they possess useful characteristics such as the extracel‑ lular secretion of the enzyme in large quantities using economical substrates (Niyonzima, 2021). Enzymes can be produced using substrates originating from agricultural byproducts. For example, maize stalks and sugarcane bagasse have been used to produce detergent-compatible cellulases by Aspergillus fungi (Imran et al., 2018; El-Baroty et al., 2019). B.R. Dave et al. (2012) used readily available, low-cost de-oiled Jatropha seed cake to obtain cellulase from Thermoascus aurantiacus RBB-1. Compared to its amorphous or mixed forms, crystalline cellulose has been observed to be a more effective carbon source for cellulase production in thermophilic fungi (Fracheboud, Ganevascini, 1989)

Fungal enzyme genes are easily cloned into bacterial strains for cellulase production, as fungal enzymes are structurally less complex than their bacterial counterparts (Maki et al., 2009; Acharya, Chaudhary, 2012). However, their primary advantage remains that cellulases can be obtained from fungi using relatively simple methods, and the fungi themselves can produce cellulases when cultivated on inexpensive substrates. Moreover, because they produce a full spectrum of cellulases, filamentous fungi remain the most popular choice for industrial cellulase production (Ilić et al., 2023).

## Bacterial cellulases

Several studies have demonstrated that bacterial cellulases possess significant advantages over fungal cellulases in specific activity and stability (Ejaz et al., 2021). Bacteria exhibit high growth rates, are adapted to diverse ecological niches, and are amenable to genetic manipulation (Nyathi et al., 2023). Numerous cellulosolytic bacterial strains have been identified, which produce specific enzymes that exhibit resistance to extreme conditions (Bhati et al., 2021). Rapid advancements in bacterial cellulase research indicate a more diverse genetic makeup than fungal cellulases, which are currently more extensively commercialised (Ilić et al., 2023)

The most common cellulolytic bacteria include Acetivibrio cellulolyticus, Bacillus spp., Cellulomonas spp., Clostridium spp., Erwinia chrysanthemi, Thermobispora bispara, Ruminococcus albus, Streptomyces spp., Thermonospora spp., and Thermobifida (Sadhu, Maiti, 2013). The search for new cellulolytic bacteria strains is currently attracting increasing attention. Consequently, numerous new species of cellulolytic bacteria have been described. These include Streptomyces abietis (Fujii et al., 2013), Kallotenue papyrolyticum (Cole et al., 2013), Ornatilinea apprima (Podosokorskaya et al., 2013), Bacteroides luti (Hatamoto et al., 2014), Alicyclobacillus cellulosilyticus (Kusube et al., 2014), Anaerobacterium chartisolvens (Horino et al., 2014), Caldicellulosiruptor changbaiensis (Bing et al., 2015), Herbinix hemicellulosilytica (Koeck et al., 2015), Pseudomonas coleopterorum (Menendez et al., 2015), Siphonobacter aquaeclarae, Cellulosimicrobium funkei, Paracoccus sulfuroxidans, Ochrobactrum cytisi, O. haematophilum, Kaistia adipata, Devosia riboflavina, Labrys neptuniae, and Citrobacter freundii (Huang et al., 2012), Thermotoga naphthophila (Akram, Haq, 2020), and Nocardiopsis dassonvillei (Sivasankar et al., 2022).

Aerobic free-living bacteria secrete extracellular enzymes equipped with binding modules for various cellulose conformations. Enzyme synergism ensures the efficient hydrolysis of cellulose-containing substrates. Anaerobic bacteria, frequently found in the gastrointestinal tracts of herbivorous animals, are characterised by an extracellular multienzyme complex like the cellulosome. Within the cellulosome, diverse cellulolytic enzymes are arranged on a scaffold protein, ensuring the secure attachment of cells to cellulose, promoting elevated local concentrations, and maintaining the proper ratios and sequence of components. The cellulosome of the thermophilic bacterium C. thermocellum was the initial subject of study, succeeded by investigations of the cellulosomes of mesophilic clostridia, ruminococci, and additional anaerobes (Schwarz, 2001).

In addition to cellulosome complexes, anaerobes, including clostridia, secrete cellulases and hemicellulases. By contrast, cellobiohydrolases have not been found in the enzyme systems of Pseudomonas, Bacillus, and Erwinia, with their function extending beyond nutrition to include facilitating the penetration of the phytopathogen into the host cell (Rabinovich et al., 2002).

## Cellulases of thermophilic microorganisms

Thermostable cellulases exhibit stability at elevated temperatures (Azadian et al., 2016). Thermostable microbial enzymes demonstrate peak functionality within a temperature range of 60 to 80 °C. In the enzymatic hydrolysis of cellulose, thermostable cellulases play a significant role as they can be used immediately following the heating stage without prior cooling, thereby reducing production cycle times and increasing yields (Liu D. et al., 2011).

Thermophilic microorganisms produce specialised proteins, referred to as chaperones, that assist in refolding proteins into their native conformation and restoring their functions (Laksanalamai, Robb, 2004; Singh S.P. et al., 2010). The small DNA-binding protein Sso7d in Sulfolobus solfataricus was observed to be involved in maintaining the stability of aggregated proteins (Ciaramella et al., 2002). However, the existence of such mechanisms suggests that enzymes from thermophilic organisms may not inherently exhibit high thermostability, especially outside of their host organism.

The high temperature tolerance of proteins derived from thermophilic bacteria, actinomycetes, and archaea is attributable to elevated electrostatic, disulfide, and hydrophobic interactions within their structural framework (Ladenstein, Ren, 2006; Pedone et al., 2008). Some thermophilic enzymes are stabilised by metal ions and inorganic salts (Vieille, Zeikus, 2001). The cellulolytic activity of certain thermophilic fungi, such as Chaetomium thermophile, Sporotrichum thermophile, and T. aurantiacus, is two to three times greater than that of Trichoderma viridae (Tansey, 1971).

Another factor that may affect thermostability is glycosylation (Kahn et al., 2020; Ramakrishnan et al., 2023). Should the bacterial gene be cloned into a protein-glycosylating organism, like fungi, this phenomenon could augment the thermostability of the enzyme

Various thermophilic bacteria, including representatives of the genera Bacillus, Geobacillus, Caldibacillus, Acidothermus, Caldocellum, and Clostridium, have been reported to produce thermostable cellulolytic enzymes (Ghosh et al., 2020). The hyperthermophilic bacterium Dictyoglomus turgidum carries a gene (Dtur_0671) encoding a β-glucosidase expressed in Escherichia coli. This enzyme exhibits maximum activity at 80 °C and pH 5.4, is extremely stable within the pH 5–8 range, and retains 70 % of its activity after 2 hours at 70 °C. The high tolerance to glucose and ethanol has proven this enzyme to be suitable for industrial bioethanol production (Fusco et al., 2018).The gene of the cellulosolytic enzyme from Thermotoga naphthophila RKU-10T was successfully obtained and expressed in E. coli. The purified enzyme, TnCel12B, was demonstrated to exhibit the maximum activity at pH 6.0 and 90 °C. It retained 100 % activity after incubation for 8 hours at 85 °C, as well as across the pH 5.0–9.0 range (Akram, Haq, 2020). A gene from the hyperthermophilic archaeon Sulfolobus shibatae, encoding endo-1,4-β-d-glucanase, demonstrated maximum activity at 95–100 °C following cloning and overexpression in E. coli. This enzyme exhibited excellent resistance to high temperatures: it retained full activity after one hour of incubation at temperatures up to 85 °C; 98, 90, and 84 % of initial activity was observed after 2 hours of incubation at 75, 80, and 85 °C, respectively (Boyce, Walsh, 2018).

Cellulases are categorised into two groups depending on their preferred pH ranges. The first group includes thermoacidophilic cellulases, such as those from Alicyclobacillus, Geobacillus, and T. aurantiacus species, that thrive in acidic conditions. The second group includes thermoalkaliphilic cellulases, such as those from Bacillus, Halobacillus, and archaea, that prefer basic conditions. Both groups of enzymes can operate effectively at high temperatures, but their application varies according to the specific requirements of different industrial sectors (Arya et al., 2024).

## Cellulases of psychrophilic microorganisms

The application of cold-active enzymes operating at alkaline pH may, for example, prove to be in demand in detergents, as it preserves the quality of fabrics without the use of hot water. Psychrophilic microorganisms are used to isolate enzymes active in the low-temperature range. Psychrophilic microorganisms represent a substantial segment of saprophytic organisms that inhabit soil, marine environments, freshwater ecosystems, and wastewater.

Metabolically active bacteria capable of surviving at temperatures between –5 and –15 °C have been isolated from Arctic permafrost (Bakermans, Skidmore, 2011; Mykytczuk et al., 2013). These psychrophiles can survive at low temperatures, with intracellular biochemical processes performed by cold-active enzymes. Furthermore, their extracellular enzymes facilitate the degradation of complex materials present in the environment. In mesophilic organisms, the impact of reduced temperatures on enzyme activity is more pronounced because their enzyme molecules possess fewer structural adaptations for functioning under such conditions, resulting in low enzymatic activity (Karan et al., 2012). Conversely, psychrophiles growing in cold environments possess cold-adapted enzymes characterised by high catalytic activity and stability

Over the past fifteen years, various psychrotrophic microorganisms have been identified. In 1996, the first low-temperature cellulase was isolated from the fungus Acremonium alcalophilum. At 40 °C and pH 7.0, this cellulase demonstrated maximum activity, with over 20 % activity retained at 0 °C (Hayashi et al., 1996). Subsequently, cellulases have been isolated and characterised from other psychrophilic microorganisms. The optimal temperature for their activity typically ranges from 20 to 40 °C (see the Table), with higher optimal temperatures occasionally reported.

**Table 1.**
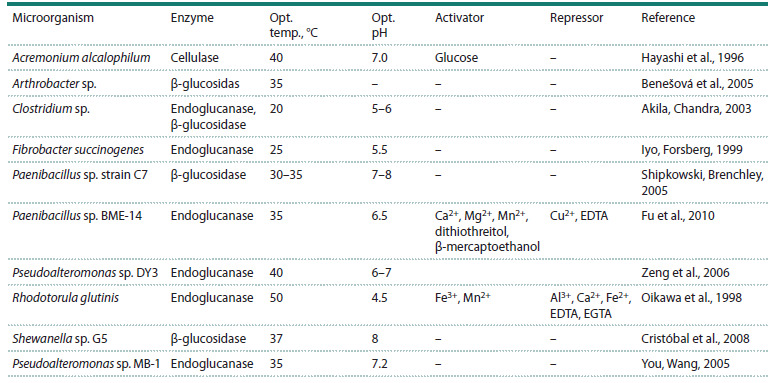
Cellulases of psychrophilic microorganisms

Microbial strains adapted to cold environments have been primarily isolated from Antarctic and polar regions. Other possible sources of cold-active cellulases are microorganisms found in mud and deep-sea sediments. A Clostridium strain isolated from manure biogas was capable of growth at temperatures from 5 to 50 °C, producing a range of xylanoand cellulolytic enzymes, which were most active at 20 °C (Akila, Chandra, 2003). Similarly, the cellulase produced by the fungus A. alcalophilum was active even at 0 °C (Hayashi et al., 1996).

CelE1, a novel cold-tolerant cellulase from the GH5 family, was isolated from a soil metagenomic library obtained from a sugarcane field. A functional screening was employed to identify cellulolytic clones within this library (Alvarez et al., 2013). The CelE1 endoglucanase isolated via this method demonstrated high activity across a broad temperature range and under alkaline conditions

T.V. Souza et al. (2015) investigated the influence of pH on the secondary and tertiary structure, charge, and activity of CelE1. While the pH change had a minimal impact on the enzyme structure, its activity diminished in acidic conditions. Optimal activity was achieved at pH 8. To assess the suitability of CelE1 as an additive in detergents and cleaning agents, its activity was evaluated in the presence of surfactants. The authors observed no significant inhibitory effect of surfactants on CelE1 endoglucanase activity (Souza et al., 2015). Furthermore, a thermodynamic analysis based on structural stability and the chemical unfolding/refolding process of CelE1 was conducted. The findings indicated that the chemical decomposition occurs through a reversible two-phase process. Thermodynamic analysis data are highly valuable in predicting enzyme stability

The psychrophilic actinobacterium Nocardiopsis dassonvillei PSY13 produces a highly active cold-adapted cellulase with optima at 10 °C and pH 7.5 (Sivasankar et al., 2022).

A cold-active cellulase (Celluzyme®) active at 15 °C has been developed by Novozymes from cold-adapted H. insolens fungi and is commercially available as part of a cellulase blend under the trade name Celluclean®.

## Cellulases of halophilic microorganisms

The universal application of cellulases necessitates the continuous search for novel enzyme sources. Enzymes of marine origin have recently attracted particular attention, especially for industrial applications. Specialised environments within the marine ecosystem, including estuaries and mangroves, are rich in lignocellulosic biomass and consequently provide a nutrient-rich habitat for cellulose-degrading organisms. Given their ability to survive in environments with limited nutrients and unfavorable circumstances, marine organisms show promise as candidates for various industrial applications (Dalmaso et al., 2015; Barzakar, 2018). The elevated salinity (3 %) of marine ecosystems, as noted by S.T. Jahromi and N. Barzakar, has resulted in a more diverse array of cellulolytic microorganisms than those found in terrestrial environments (Jahromi, Barzakar, 2018).

Investigations into cellulose degradation in the presence of salt have identified new metabolic pathways and enzymes that exhibit cellulolytic activity. Cellulose-degrading organisms in marine ecosystems significantly contribute to the mineralization of organic matter, which enhances the productivity of these ecosystems (Milici et al., 2017). The capacity to degrade cellulose has been observed across various marine organisms, including bacteria (Harshvardhan et al., 2013), yeasts (Rong et al., 2015), filamentous fungi (Liu J. et al., 2012; Batista-García et al., 2017), protists (Bremer, Tabbot, 1995), rotifers (Chun et al., 2997), krill (Tsuji et al., 2012), and echinoderms (Sakamoto et al., 2007).

A halotolerant endoglucanase with a molecular weight of 39 kDa was isolated from the filamentous fungus Botrytis ricini URM 5627 (Silva et al., 2018). The optimal operating conditions for the enzyme were 50 °C and pH 5. The enzyme remained stable at 39–60 °C for 60 minutes and at pH 4–6. Enzymatic activity was observed to increase in the presence of Na+, Mn2+, Mg2+, and Zn2+ and decrease in the presence of Ca2+, Cu2+, and Fe2+. The endoglucanase exhibited a halotolerant profile, with its activity increasing proportionally with NaCl concentration. The highest activity was observed at 2 M NaCl, representing a 75 % increase.

## Selection of producers most suitable for enzyme production

E. coli and Bacillus sp. are widely used as bacterial systems for recombinant protein expression. In addition, other bacteria, including Zymomonas mobilis and Streptomyces lividans, are also used as platforms. In the industry, E. coli is the most commonly employed cellulase expression system, possessing several benefits, including a thoroughly characterised genome, commercial availability, and ease of modification. However, certain drawbacks have to be considered, including limited secretion (due to the thick outer membrane hindering transport across the cell membrane), degradation of linker sequences, reduced cellulolytic activity, and the potential for inclusion body formation. Zymomonas mobilis has proven to be an alternative to yeast due to its versatility in fermenting a wide range of sugars. Moreover, it serves as an alternative to E. coli due to its capability to express recombinant proteins both intracellularly and extracellularly.

Genetic methodologies for C. thermocellum are less advanced than those for model organisms like E. coli, and the introduction of single nucleotide polymorphisms (SNPs) has received limited attention. C. thermocellum is an obligate thermophilic and anaerobic Gram-positive bacterium that naturally ferments lignocellulose into ethanol and organic acids (Lynd et al., 2005; Olson et al., 2012; Xu Q. et al., 2016; Tian et al., 2019).

There are two main strategies for improving cellulases or components of the cellulase complex through genetic modifications: (1) rational design and (2) directed evolution (Acharya, Chaudhary, 2012). The merits and modifications (classic random mutagenesis or genetic modification) of each approach are tested and employed by various specialists to achieve maximum cellulase yield and efficiency. To obtain the maximum cellulase yield, S. Sadhu et al. performed random mutagenesis on a cellulolytic strain of the genus Bacillus using N-methyl-N′-nitro-N-nitrosoguanidine (NTG), resulting in AT and GC transition mutations (Sadhu et al., 2014). This yielded a mutant strain with enhanced carboxymethylcellulase activity. Similar results were obtained using NTG on Cellulomonas sp. (Sangkharak et al., 2012), but the characteristics of these mutants have not been reported.

The production of recombinant enzymes involves developing technologies that combine directed evolution and rational design (Zhao H. et al., 2002; Cherry, Fidantsef, 2003). Nonetheless, a significant barrier to rational design stems from incomplete understanding of cellulase substrates, their enzymatic interactions, interrelationships, and the regulation of cellulase activity, often resulting in the prevalent application of directed evolution (Zhang Y.H.P. et al., 2006).

Nevertheless, there are examples of successful application of the rational design method. In a study by A. Akbarzadeh et al. (2018), directed mutagenesis was employed on endoglucanase-II (Cel5A) derived from H. jecorina. This enzyme demonstrates thermal instability due to the presence of four disulfide bonds in its structure. Cysteine amino acid residues at positions 99 and 323 were substituted with valine and histidine, respectively. The loss of two disulfide bonds resulted in increased activity and thermostability of the enzyme. The activity of cellulase from Gloeophyllum trabeum (GtCel5) was enhanced using site-directed mutagenesis of loop 6 (Zheng et al., 2018). A.S. Dotsenko et al. (2020) demonstrated that the thermostability of cellobiohydrolase could be improved using rational design by substituting proline. The resulting G415P protein exhibited a 3.5-fold increase in half-life at 60 °C compared to the wild-type protein. A number of authors have reviewed and compared the expression systems of recombinant cellulases (Garvey et al., 2013; Hasunuma et al., 2013; Sadhu, Maiti, 2013; Juturu, Wu, 2014; Lambertz et al., 2014).

Several studies have documented the employment of directed evolution techniques in conjunction with rational design to achieve cellulase overexpression within their native bacterial hosts. Ease of genetic modification and other attributes have allowed species such as B. subtilis and C. thermocellum to be used as homologous cellulase production systems.

Nevertheless, employing these bacteria presents drawbacks, including limited protein production, elevated manufacturing expenses, and the necessity of culture amplification in an enriched environment (Lambertz et al., 2014). The use of an E. coli strain for the expression of β-1,4-endoglucanase and β-1,4-glucosidase from another E. coli strain under a constitutive promoter was reported, which allowed for biomass hydrolysates fermentation (Munjal et al., 2015).

D. Chung et al. (2014) engineered Caldicellulosiruptor bescii, a bacterium capable of independently degrading lignocellulosic biomass. The study involved the homologous expression and cloning of a multimodular cellulase, CelA, which consists of GH9 and GH48 domains.

A considerable number of studies have also focused on applying the classical method of overproducing the target protein or enzyme by cloning its coding genes into a high copy-number plasmid. For example, this method was used to obtain homologous overexpression of cellulase CelC2 from Rhizobium leguminosarum bv. trifolii ANU843, which increased its cellulolytic activity 3-fold (Robledo et al., 2011).

An effective Agrobacterium tumefaciens-mediated transformation system for H. insolens was developed by X. Xu et al. (2016). The authors transformed plasmids carrying the H. insolens glyceraldehyde-3-phosphate dehydrogenase gene promoter, which controlled the transcription of genes encoding neomycin phosphotransferase, hygromycin B phosphotransferase, and enhanced green fluorescent protein. T-DNA insertional mutagenesis was used to create a mutant library of H. insolens. As a result, a transformant identified as T4, exhibiting elevated cellulase and hemicellulase activity, was isolated. The activities of phospholipase, endoglucanase, cellobiohydrolase, β-glucosidase, and T4 xylanase at the fermentation endpoint exceeded those of the wild-type strain by 60, 440, 320, 41, and 81 %, respectively.

Strategies based on heterologous expression focus on employing non-cellulolytic microorganisms with high production rates for the expression of microbial cellulases (Bhattacharya et al., 2015). In both research and industry, most frequently used are bacteria such as E. coli, various species of the genera Bacillus, Pseudomonas fluorescens, Ralstonia eutropha, and Zymomonas mobilis, yeasts such as Saccharomyces cerevisiae and Pichia pastoris, and mycelial fungi from the genera Aspergillus and Trichoderma. Furthermore, mammalian, plant, or insect cell cultures, as well as transgenic plants and/or animals, are employed for protein expression (Demain, Vaishnav, 2009).

## Conclusion

Due to their wide-ranging applications in cellulose-degrading biocatalytic processes, cellulase enzymes have seen an increase in industrial demand over the last few years. The broad applicability and environmental compatibility of cellulase-mediated processes continue to drive research aimed at discovering efficient and cost-effective enzymes.

Filamentous fungi cultures have traditionally been employed in cellulase production. However, most filamentous fungi obtained through natural selection exhibit low secretory capacity for cellulase production, which is insufficient to meet industrial demands.

An effective method for increasing fungal enzyme production is random mutagenesis combined with an adaptive laboratory evolution strategy (Peng et al., 2021). In recent years, research has increasingly focused on bacterial cellulases, due to their diverse properties, enhanced stability, and the potential to integrate multiple activities within a single enzyme.

Extremophilic microorganisms possess a variety of molecular strategies for surviving extreme conditions. Their enzymes exhibit properties such as salt tolerance, thermostability, and cold adaptiveness. Enzymes that are thermophilic, piezophilic, acidophilic and halophilic have been isolated recently. Through the employment of genetic engineering, these genes can be expressed in other organisms, leveraging an extensive array of existing operational methodologies.

Cellulase immobilization technologies, especially the use of the combination of polymeric carriers with nanomaterials, have attracted considerable attention. It is possible for such immobilised cellulases to exhibit enhanced activity, stability, reusability, and processability. The combination of nanomaterials and biocatalysis technologies using immobilised cellulases is currently considered a cutting-edge field of research and development in enzyme technology (Ranjan et al., 2023).

The complexity of cellulase presents a unique challenge. All three components of the enzyme complex are essential for its proper functioning (Bhat M.K., Bhat S., 1997). This complex nature makes it difficult to clone the enzyme into heterologous systems. Consequently, significant global research efforts are focused on identifying natural producers and devising strategies to enhance the properties of cellulase through genetic modification of isolated organisms. Individual cellulase complex components are cloned and expressed using standard production systems employed in modern biotechnology.

## Conflict of interest

The authors declare no conflict of interest.
